# The Hospital Officer's Bookshelf

**Published:** 1912-07-13

**Authors:** 


### THE HOSPITAL OFFICER'S BOOKSHELF.

Scientific Features of Modern Medicine. By Frederic S. Lee, Ph.D. (The Columbia University Press. 1911. Pp. 186. Price 6s. 6d. net. Agents: Henry Frowde and Co.)

This pleasant gossipy book is a reprint of the Jesup Lectures at Columbia University, and is of the nature of an exposition of medical and surgical principles for popular consumption. The language used is accordingly not too technical, and the author has succeeded well in his attempt to present the facts in such a form as to be clearly comprehensible to any intelligent layman. Throughout the book the scientific aspects of modern medical and surgical thought are explained, and the reason is shown for many of those mysterious processes by which medical men assist themselves to form their diagnoses. In short, the book is an excellent antidote for lay faddisms of all kinds, especially for "Christian science," and faith-healers. The only objection that will be raised is in respect of the high price, which will certainly restrict the circulation in this country. At two shillings or half-a-crown it might reach a large public, but, as it is, probably few people will buy it.

Diseases of the Nervous System. By Judson S. Bury, M.D., F.R.C.P. Manchester University Medical Series, No. XIV. (Sherratt and Hughes. 1912. Pp. 778. Price 15s. net.)

The output of text-books upon diseases of the nervous system seems to be for some reason smaller than that upon some other special departments of medicine and surgery—gynæcology, for example, or anæsthetics, or infant feeding. There are, it is true, two or three really excellent works published; but the most satisfactory of them are either encyclopædic in scope, or else elementary guides for medical students. Of text-books about the size of this and suited so exactly to the needs of the house physician or the intelligent general practitioner, there are none which can be said to surpass this excellent book by Dr. Judson Bury. Medical students, too, who are always inclined to regard nervous diseases (and skin diseases) as mysterious provinces of medicine too hard for ordinary mortals to explore, will find that the logical arrangement and lucid teachings of this book will solve many of their difficulties in understanding the subject. The book is copiously illustrated, and photographs of actual patients showing some particular paralysis or attitude are especially numerous. It is excellently printed and produced, and is written in a pleasant and graceful style, which makes it very easy reading. Altogether it is a work which should attain wide and rapid popularity; for it can be thoroughly recommended as a guide to the often complicated and obscure diseases with which it deals. It is of the very best class of British medical text-book, and we know of no higher encomium than that to bestow upon it.

Modern Views upon the Significance of Skin Eruptions. By H. G. Adamson, M.D. The Goulstonian Lectures for 1912. (London: J. Bale, Sons, and Danielsson. Pp. 103. Price 3s. 6d. net.)

This scholarly written and beautifully illustrated essay —the first Goulstonian lecture allotted to a dermatologist since 1873—presents in an eminently readable form an epitome of all that is most stimulating in recent progress in dermatology; and by stimulating we mean most suggestive of the lines of further advance. It bears the unmistakable imprint of careful preparation, wide erudition, and breadth of view. Nor is the interest of it only for dermatologists; it is bound to appeal also to all physicians, for it deals with much more than the classification of rashes and the prescribing of ointments. The selection of Dr. Adamson to deliver these lectures was, in fact, a most judicious one, and has been a pronounced success; we congratulate him both upon the honour and the performance.

